# Supplementary material for: Unveiling Misconceptions among Small-Scale Farmers Regarding Ticks and Tick-Borne Diseases in Balochistan, Pakistan
Source: Vet Sci. 2024 Oct 12;11(10):497. doi: 10.3390/vetsci11100497 (PMC11512219; doi:10.3390/vetsci11100497)
Supplement: Supplementary file 1 [file vetsci-11-00497-s001.zip › Table S1.pdf]

Supplementary material. Questionnaire on knowledge, attitude and practices to evaluate knowledge, attitude and practices among farmers of grazing animals.

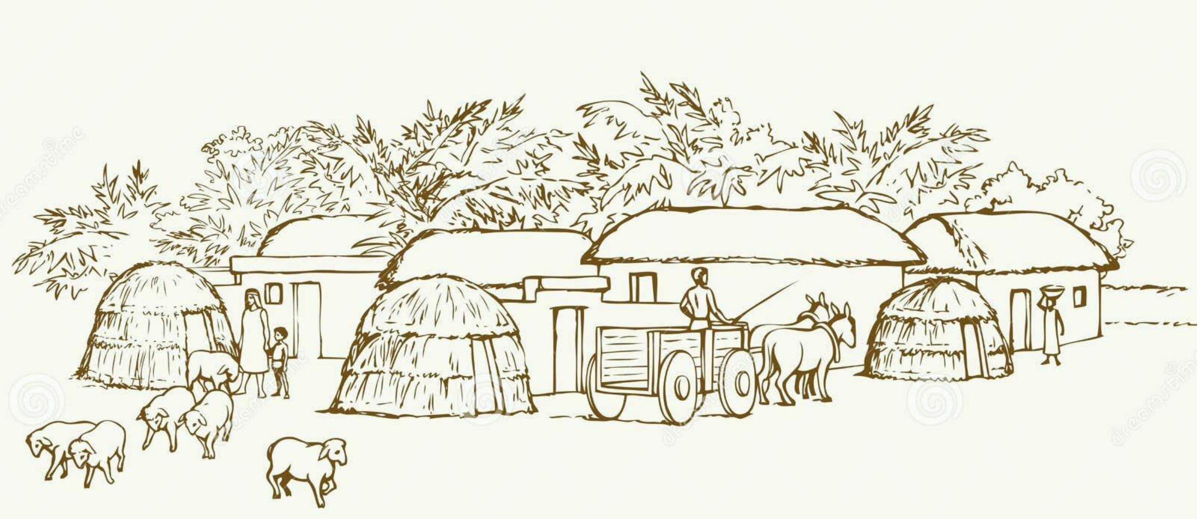

Sociodemographic characters of farms

Name of the farmer________________________

District____________________

Date of interview __________________________

Language used for interview_________________

Coordinate of study site _____________________

Q. No.	Question	Options
A1	What is your age?	18-24, 25-45, 45-60, > 60
A2	What is your marital status?	Single, Married
A3	Please specify your ethnicity.	Pashtoon, Baloch, Others
A4	What is your level of urbanicity?	Urban, Rural
A5	What is your qualification?	Illiterate, Primary education, Secondary education, College and above
A6	How many years of experience do you have in dealing with animals?	< 5, 5-10, 10-15, > 15
A7	What is your monthly income?	> $200, $200-300, > $300
A8	What kind of grazing animals do you have?	Goats, Sheep, Cattle, Buffalo, Camels, Horses
A9	Do you have any pets along with your grazing animals?	Dog, Cat, Rabbit

Knowledge related questions

Q. No.	Question	Options
B1	Ticks are present on our grazing animals.	I agree, I do not agree, I do not know
B2	Ticks are different from insects.	I agree, I do not agree, I do not know
B3	Ticks can transmit tick-borne diseases?	I agree, I do not agree, I do not know
B4	The life of a tick consists of four stages.	I agree, I do not agree, I do not know
B5	Ticks can jump onto the host.	I agree, I do not agree, I do not know
B6	Tick-borne diseases can be cured with antibiotics.	I agree, I do not agree, I do not know
B7	Climate change can impact the tick population.	I agree, I do not agree, I do not know
B8	Ticks have an economic impact on grazing animals.	I agree, I do not agree, I do not know
B9	An effective tick-borne vaccine is available.	I agree, I do not agree, I do not know
B10	Performing tick-checks can prevent TBDs.	I agree, I do not agree, I do not know

Attitude related questions

Q. No.	Question	Yes	No
C1	Are TBDs a serious problem?		
C2	Concerns about tick bites and tick-borne infections.		
C3	Spraying is the best method to reduce ticks?		
C4	Move animals to highland pastures during summer.		
C5	Provide medical treatment to infested animals.		
C6	Sell highly infested animals.		
C7	Grazing animals have access to food and clean water.		
C8	Separate infested animals from healthy ones.		
C9	Limit children's interaction with grazing animals		

Practices related questions

Q. No.	Questions	Yes	No
D1	Participate in tick control training sessions		
D2	Engage with local farmer community		
D3	Use acaricides according to the manufacturer's guidelines		
D4	Receive government subsidies on acaricides		
D5	Avoid typical tick habitats		
D6	Tuck pants into socks or boots		
D7	Wear light-coloured clothing as preventive measures		
D8	Check body for ticks upon return from outdoors		
D9	Shower immediately after returning indoors		

کردار آبادیاتی سماجی کے فارموں

نام کا کسان

ضلع

تاریخ کی انٹرویو

زبان والی ہونے استعمال لیے کے انٹرویو

کوآرڈینیٹ کا جگہ کی مطالعہ

نمبر سوال	سوال	اختیار
A1	ہے؟ کیا عمر کی آپ	18-24، 25-45، 45-60، > 60
A2	ہے؟ کیا حیثیت ازواجی موجودہ کی آپ	شدہ شادی ،شدہ شادی غیر
A3	ہے؟ سے قوم کس تعلق کا آپ	دیگر ،بلوچ ،پشتون
A4	ہے؟ کیا سطح کی شہریت کی آپ	دیہی ،شہری
A5	ہے؟ کیا قابلیت تعلیمی کی آپ	یا ،تعلیم ثانوی ،تعلیم پرائمری ،ناخواندہ زیاد
A6	ہے؟ تجربہ کا سالوں کتنے کا نمٹنے سے جانوروں کو آپ	<5، 5-10، 10-15، > 15
A7	ہے؟ کیا آمدنی ماہانہ کی آپ	> $200، $200-300، > $300
A8	ہیں؟ جانور والے چرنے سے کون پاس کے آپ	،اونٹ ،بھینس ،گائے ،بھیڑ ،بکریاں گھوڑے
A9	جانور پالتو کوئی ساتھ کے جانوروں والے چرنے اپنے پاس کے آپ کیا ہے؟	خرگوش ،بلی ،کتا

سوالات سے متعلق علم

نمبر سوال	سوال	اختیار
B1	ہیں۔ ہوتے موجود چچڑ پر جانوروں والے چرنے	نہیں میں ،ہوں نہیں متفق میں ،ہوں متفق میں جانتا
B2	ہیں۔ ہوتی مختلف سے کیڑوں چچڑ	نہیں میں ،ہوں نہیں متفق میں ،ہوں متفق میں جانتا
B3	ہیں؟ سکتے کر منتقل کو بیماریوں چچڑ	نہیں میں ،ہوں نہیں متفق میں ،ہوں متفق میں جانتا
B4	ہے۔ ہوتی مشتمل پر مراحل چار زندگی چچڑکی	نہیں میں ،ہوں نہیں متفق میں ،ہوں متفق میں جانتا
B5	ہیں۔ سکتے کود پر میزبان چچڑ	نہیں میں ،ہوں نہیں متفق میں ،ہوں متفق میں جانتا

B6	ہے۔ سکتا جا کیا سے بائیوٹک اینٹی علاج کا بیماریوں والی ہونے پیدا سے چچڑ	نہیں میں ،ہوں نہیں متفق میں ،ہوں متفق میں جانتا
B7	ہے۔ سکتی کر متاثر کو آبادی چچڑ تبدیلی موسمیاتی	نہیں میں ،ہوں نہیں متفق میں ،ہوں متفق میں جانتا
B8	ہے۔ پڑتا اثر معاشی پر جانوروں والے چرنے کے چچڑ	نہیں میں ،ہوں نہیں متفق میں ،ہوں متفق میں جانتا
B9	ہے۔ دستیاب ویکسین موثر لئے کے چچڑ	نہیں میں ،ہوں نہیں متفق میں ،ہوں متفق میں جانتا
B10	ہے دستیاب ویکسین لئے کے کرنے چیک کو چچڑ	نہیں میں ،ہوں نہیں متفق میں ،ہوں متفق میں جانتا

سوالات سے متعلق رویوں

نمبر سوال	سوال	اختیار
C1	ہیں؟ مسئلہ سنگین ایک بیماریاں والی ہونے پیدا سے چچڑ کیا □	نہیں / ہاں
C2	بارے کے انفیکشن والے ہونے پیدا سے اس اور کاٹنے کے چچڑ کو آپ کیا □ ہیں؟ خدشات میں	نہیں / ہاں
C3	ہے؟ کرنا سپرے طریقہ بہترین کا کرنے کم کو چچڑ کیا □	نہیں / ہاں
C4	ہیں؟ کرتے منتقل میں چراگاہوں اونچی کو جانوروں میں گرما موسم آپ کیا □	نہیں / ہاں
C5	ہیں؟ کرتے فراہم علاج طبی کو جانوروں متاثرہ آپ کیا □	نہیں / ہاں
C6	ہیں؟ کرتے فروخت جانور متاثرہ زیادہ آپ کیا □	نہیں / ہاں
C7	ہے؟ حاصل رسائی تک پانی صاف اور خوراک کو جانوروں والے چرنے کیا □	نہیں / ہاں
C8	ہیں؟ کرتے الگ سے جانوروں مند صحت کو جانوروں متاثرہ آپ کیا □	نہیں / ہاں
C9	ہیں؟ کرتے محدود کو تعامل کے بچوں ساتھ کے جانوروں والے چرنے آپ کیا □	نہیں / ہاں

سوالات سے متعلق مشقوں

نمبر سوال	سوال	اختیار
D1	ہیں۔ لیتے حصہ میں سیشنز تربیتی کے کنٹرول کے چچڑوں	نہیں / ہاں
D2	ہیں۔ رہتے مشغول ساتھ کے کمیونٹی کی کسانوں مقامی	نہیں / ہاں
D3	ہیں۔ کرتے استعمال کا ایکاریسائیڈز مطابق کے ہدایات کی مینوفیکچرر	نہیں / ہاں
D4	ہیں؟ کرتے حاصل سبسڈی حکومتی پر ایکاریسائیڈز	نہیں / ہاں
D5	ہیں۔ بچتے سے مسکن کے چچڑوں عام	نہیں / ہاں
D6	ہیں؟ کرتے اندر کے جوتوں یا جرابوں شلوارکو	نہیں / ہاں
D7	ہیں؟ پہنتے کپڑے کے رنگ ہلکے پر طور کے تدابیر احتیاطی	نہیں / ہاں
D8	ہیں؟ کرتے چیک لئے کے چچڑوں کو جسم پر واپسی سے باہر	نہیں / ہاں
D9	ہیں؟ لیتے شاور فوراً پر واپسی گھر	نہیں / ہاں
