# Supplementary figures and images for: Unveiling Misconceptions among Small-Scale Farmers Regarding Ticks and Tick-Borne Diseases in Balochistan, Pakistan
Source: Vet Sci. 2024 Oct 12;11(10):497. doi: 10.3390/vetsci11100497 (PMC11512219; doi:10.3390/vetsci11100497)

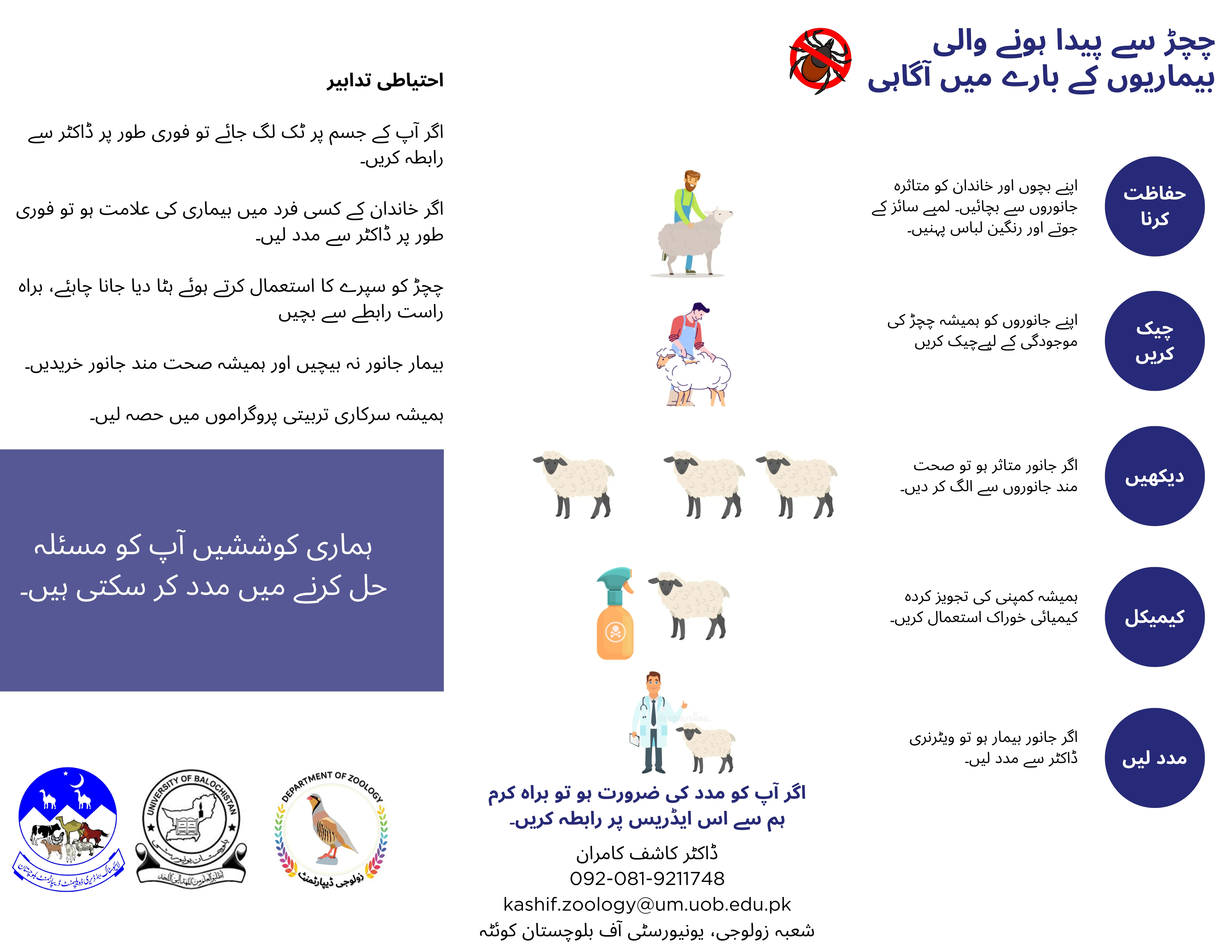

Supplement: Supplementary file 1 [file vetsci-11-00497-s001.zip › Figure S1.tiff]
